# The effect of sleep disturbance on the association between work–family conflict and burnout in nurses: a cross-sectional study from South Korea

**DOI:** 10.1186/s12912-022-01114-7

**Published:** 2022-12-12

**Authors:** Sujeong Han, Sungjung Kwak

**Affiliations:** 1 College of Nursing, Konyang University, Daejeon, Republic of Korea; 2 Robotic Surgery Center, Konyang University Hospital, 158 Gwanjeodong-ro, Seo-gu, Daejeon, 35365 Republic of Korea

**Keywords:** Burnout, Family conflict, Nurses, Sleep disorder, Work

## Abstract

**Background:**

Sleep disturbances can lead to work–family conflicts and affect the mental health of nurses. This study aimed to investigate the mediating effect of sleep disturbance on the association between work–family conflict (WFC) and burnout in nurses.

**Methods:**

Responses to a questionnaire from 156 nurses working in a hospital in South Korea were analyzed. Multiple linear regression analysis and PROCESS Macro Model 4 were used to analyze the mediating effect of sleep disturbance on the relationship between WFC and burnout. A bootstrapping approach was used to test the statistical significance of the indirect parameter effects.

**Results:**

The WFC of nurses had a positive correlation with sleep disturbance and burnout. Moreover, sleep disturbance completely mediated the association between WFC and burnout.

**Conclusions:**

Nursing administrators should pay careful attention to WFCs that interfere with nurses’ sleep and reduce their sleep quality, and design suitable working schedules that minimize the effects of WFC. In addition, hospital administrators should improve shift scheduling to ensure good sleep quality and reduce the health effects of WFC among nurses.

## Background

Burnout is caused by excessive stress at work and characterized by emotional fatigue, depersonalization, and decreased personal achievement [1]. The incidence of burnout is higher in professional occupations than in the general population [2] and has been reported among nurses in several countries. In particular, researchers have been actively conducting research on burnout incidence rates, risk factors, and effects among nurses under conditions such as the COVID-19 pandemic. In the United States, a study described the prevalence of burnout among nurses as ranging from 35 to 45% [3]; in a large Chinese study, female nurses had the highest levels of burnout-related depression, anxiety, insomnia, and distress [4]. Burnout in nurses lowers their job performance and care delivery quality, threatens patient safety (e.g., due to medication errors, falls, and infections), and increases turnover intention [1].

Previous studies have shown an association between work–family conflict (WFC) and burnout, particularly pertaining to emotional fatigue, in nurses [5]. Nurses may experience burnout due to WFC, as it threatens positive resources, such as their psychological and physical health [6, 7]. Greenhaus and Beutell [8] extended the theory of roles to define WFC as a form of role conflict that occurs when the burdens and responsibilities of work and family are incompatible. In other words, because of the specific tasks required by other roles, it is difficult to adequately fulfill tasks in one role. Research shows that nurses are experiencing an increasing incidence of WFC [7]; this is an important issue for these professionals due to their demanding working conditions, which include heavy shift work, physical or emotional workloads, and rigorous patient monitoring [9].

Regarding the topic of WFC and sleep, studies have shown the following: WFC is associated with the quantity and quality of sleep [10]; the higher the WFC, the higher the sleep deprivation of nurses [11]; higher levels of WFC significantly increase sleep disturbances [12]. Sleep disturbance is a classic health problem among nurses that is mostly caused by irregular sleep patterns owing to their shift work; the fewer sleep problems nurses have, the better their perceived health status [13]. Moreover, sleep disturbances in nurses are significant indicators of fatigue and burnout due to the physical and psychological symptoms that they evoke [14]. Although WFC and sleep disorders are related to burnout among nurses and should be researched further, to the best of our knowledge, no studies on the relationship between WFC and sleep disorders in Korean nurses have been conducted.

In Korea, the remnants of traditional gender roles emphasize women’s responsibility for housework and child-rearing. According to a survey by the Korea Institute for Health and Social Affairs [15], Korean women spend 7.4 and 3.5 times more time on housework and childcare, respectively, than do men. Nonetheless, as women’s participation in economic activities increases, their roles in work and family gradually become incompatible because performing these roles concomitantly may cause overload; this makes the finding that the reasons for nurses’ turnover are related to marriage, childbirth, and child-rearing problems unsurprising [16]. As aforementioned, the traditional gender role for women in Korea stresses on the impeccable management of family-related matters, even if they work. Accordingly, married women in Korea may suffer from WFC due to the triggering of negative emotions related to the following situation: they compulsively aim for impeccability at both work and home and subsequently experience frustration for the unfulfillment of this desire for “perfection.” As described above, nurses often do not get enough sleep due to shift work and are highly likely to have poor sleep quality and quantity due to changes in sleep rhythm [10]. Thus, it appears that Korean women do not get enough sleep due to housework and childcare.

Following these depictions in the literature, research on work–family reconciliation and sleep disorders is needed for nurses to experience reduced WFC, maintain work–family harmony and balance, and create synergistic effects. However, factual and objective research data on what Korean nurses are actually experiencing remain insufficient. Therefore, using survey data, this study aimed to investigate the mediating effect of sleep disturbance on the association between WFC and burnout in nurses. We hope that our findings will help nursing managers to flexibly adjust nurses’ work schedules in order to avoid the exacerbating effects of WFC and sleep disturbances on nurses, and in turn, patients and organizations.

## Methods

### Study design

This study used a cross-sectional design to determine the relationships among sleep disturbances, WFC, and burnout, as well as understand the mediating effect of sleep disturbance on the relationship between WFC and burnout, using questionnaire survey data, in registered nurses.

### Participants

The participants were nurses working at a university hospital (surgery, internal medicine, orthopedic surgery, obstetrics and gynecology, and pediatrics) located in D city, South Korea. Of 169 individuals from whom data were collected, Excluding 13 who did not all respond to questions and data from 156 individuals were finally analyzed. To ensure an appropriate sample size for the linear multiple regression analyses, G*power version 3.1.9.2 was used; considering a significance level of .05, a median effect size of .15, a power of 95%, and two predictors, the sample size was calculated to be 107 participants. Our final sample met this requirement. It was estimated that participants would take 10 minutes to complete the questionnaire.

### Measures

Burnout and sleep disturbance were measured using the Copenhagen Psychosocial Questionnaire II (COPSOQ II) [17]. We used the Korean version developed by June and Choi [18], which had good construct validity, as verified by Choi [19]. Each item for burnout (six questions) and sleep disturbance (four questions) was measured on a five-point Likert scale (1, not at all; 2, a small part of the time; 3, part of the time; 4, a large part of the time; 5, all the time). The items measuring the dimensions with five response categories were scored as 0, 25, 50, 75, and 100; these were calculated as the mean of the item responses, with higher scores indicating higher levels of burnout and sleep disturbance. In this study, Cronbach’s alpha values of the burnout and sleep disturbance scales were 0.85 and 0.91, respectively.

WFC was measured using the 10-item Work-Family Conflict Scale developed by Song et al. [20], which was responded to on a five-point Likert scale, with higher combined scores indicating higher levels of WFC. The Cronbach’s alpha value was 0.87 in Choi’s [19] and Lee and Kang’s [21] studies, and 0.86 in this study.

### Data analysis

We analyzed the data using SPSS version 20 and used descriptive statistics to estimate participants’ general characteristics and the mean of the variables (Tables 1 and 2). Differences in burnout according to general characteristics were analyzed using t-tests and analysis of variance (ANOVA). When the difference between groups was significant, a multiple comparison post-hoc test was performed using Scheffé’s test (Table 1). We calculated the correlations between burnout, WFC, and sleep disturbance using Pearson’s correlation coefficient (Table 3). Finally, to explore the mediating effect of sleep disturbance on the association between WFC and burnout in nurses, we conducted regression analysis using Hayes’s PROCESS Macro Model 4 [22] (Table 4). We used a bootstrapping approach for the statistical significance test of the parametric indirect effect, with the bootstrap resampling number set to 10,000 and a bias-corrected 95% confidence interval (CI) estimated and tested. Bootstrapping is widely used to test mediating effects by reducing errors caused by the assumption of normality in the distribution of indirect effects in the existing Sobel test [23].

**Table 1:** Characteristics of participants and variable differences according to general characteristics

Variable		N (%)	Burnout	WFC	SD
			M ± SD		
Gender					
	t or F(p)		3.439 (0.001)	−.181 (0.857)	1.123 (0.263)
Female		144 (92.3)	79.65 ± 10.13	28.79 ± 6.76	71.45 ± 16.48
Male		12 (7.7)	68.88 ± 13.58	29.16 ± 8.41	65.83 ± 18.92
Age (years)			28.62 ± 5.47 (years)		
	t or F(p)		1.589 (0.180)	1.493 (0.207)	3.092 (0.018)
≥ 25		49 (31.4)	78.97 ± 10.14	28.32 ± 7.06	74.08 ± 15.46
26–30		71 (45.5)	80.51 ± 11.85	29.43 ± 7.08	72.81 ± 16.62
31–35		15 (9.6)	76.88 ± 6.72	30.06 ± 5.99	67.33 ± 14.86
36–40		13 (9.3)	73.84 ± 11.20	29.07 ± 5.40	61.53 ± 13.90
≥ 41		8 (5.1)	74.58 ± 9.91	23.62 ± 6.41	58.75 ± 22.79
Marital status					
	t or F(p)		3.439 (0.001)	−0.181 (0.857)	1.123 (0.263)
Unmarried		29 (18.6)	79.65 ± 10.13	28.79 ± 6.76	71.45 ± 16.48
Married		127 (81.4)	68.88 ± 13.58	29.16 ± 8.41	65.83 ± 18.92
Education					
	t or F(p)		0.247 (0.781)	0.115 (0.892)	1.491 (0.228)
Associate		21 (13.5)	80.31 ± 10.94	28.28 ± 6.76	68.33 ± 21.46
Bachelor’s		121 (77.5)	78.65 ± 10.96	28.81 ± 6.92	72.19 ± 16.04
Master’s or higher		14 (9.0)	78.09 ± 9.21	28.14 ± 7.0	85.00 ± 13.00
Work experience (years)			63.87 ± 5.47 (months)		
	t or F(p)		4.063 (0.004)	0.095 (0.984)	3.033 (0.019)
< 1 ^a^		24 (15.4)	73.47 ± 12.21	28.29 ± 6.63	70.20 ± 17.22
1 ~ < 3 ^b^		52 (33.3)	81.15 ± 9.58	28.92 ± 7.50	74.80 ± 14.71
3 ~ < 5 ^c^		23 (14.7)	83.18 ± 9.87	29.08 ± 6.68	76.08 ± 15.51
5 ~ < 10 ^d^		28 (17.9)	79.04 ± 10.91	28.60 ± 7.43	68.38 ± 17.37
≥ 10 ^e^		29 (18.6)	75.48 ± 9.93 c > ^a^	28.24 ± 5.85	66.44 ± 17.53
Unit type					
	t or F(p)		0.986(0.401)	2.148(0.097)	3.150(0.027)
Med-surgical ward^a^		71 (45.5)	79.85 ± 9.84	29.80 ± 6.67	72.81 ± 14.99
Intensive care unit^b^		65 (41.7)	78.25 ± 11.88	28.53 ± 6.95	71.07 ± 17.90
Outpatient department^c^		10 (6.4)	74.00 ± 10.51	28.40 ± 5.87	56.00 ± 15.05
Comprehensive care ward^d^		10 (6.4)	80.20 ± 9.81	24.10 ± 7.50	73.00 ± 16.02
Current position					
	t or F(p)		2.339 (0.021)	1.271 (0.206)	3.466 (0.001)
Staff nurse		139 (89.1)	79.52 ± 10.59	29.06 ± 6.90	72.58 ± 15.98
Charge nurse or higher		17 (10.9)	73.13 ± 10.89	26.82 ± 6.52	58.23 ± 17.22
Shift work					
	t or F(p)		2.204 (0.029)	1.745 (0.083)	4.129 (<.001)
8-hour		125 (80.1)	79.76 ± 10.58	29.29 ± 6.89	73.64 ± 15.68
12-hour		31 (19.9)	75.05 ± 10.88	26.90 ± 6.58	60.48 ± 16.65

*M* Mean, *SD* Standard deviation, *SD* Sleep disturbance, *WFC* Work–family conflict

^a,b,c,d,e^Results of multiple comparison post hoc tests using the Scheffé test; the burnout of 3 to 5 years of work experience is greater than that of less than 1 year of work experience

**Table 2:** Descriptive statistics of burnout, work–family conflict, and sleep disturbance

Variable	Minimum	Maximum	M ± SD
Burnout	36.67	100.00	78.82 ± 10.77
Work–family conflict	10.00	50.00	28.82 ± 6.87
Sleep disturbance	20.00	100.00	71.02 ± 16.68

*M* Mean, *SD* Standard deviation

**Table 3:** Correlation coefficients among burnout, work–family conflict, and sleep disturbance

Variable	Burnout	Work–family conflict	Sleep disturbance
	r (p)		
Burnout	1	–	–
Work–family conflict	0.224 (0.005)	1	–
Sleep disturbance	0.500 (< 0.001)	0.223 (0.005)	1

**Table 4:** Mediating effect of sleep disturbance on the association between work–family conflict and burnout

DV	IV	Coefficient	se	t	P	LLCI	ULCI	F(p)	R^**2**^
SD	Constant	55.45	5.64	9.82	< 0.001	44.30	66.60	8.04	0.05
	WFC	0.54	0.19	2.83	0.005	0.16	0.91	(0.005)	
BO	Constant	51.77	4.10	12.60	< 0.001	43.65	59.89	27.29	0.26
	WFC	0.19	0.11	1.66	0.099	−0.03	0.20	(<.001)	
	SD	0.31	0.05	6.64	< 0.001	0.21	0.39		
BO	Constant	68.72	3.64	18.85	< 0.001	61.527	75.92	8.11	0.05
	WFC	0.35	0.12	2.84	0.005	0.10	0.59	(0.005)	
Indirect effect WFC → SD → BO		0.17	0.08			0.01	0.32		

Bootstrap sample size = 10,000

*DV* Dependent variable, *IV* Independent variable, *ULCI* Upper limit of confidence interval, *LLCI* lower limit of confidence interval, *SD* Sleep disturbance, *BO* Burnout, *WFC* Work–family conflict

## Results

### Demographics of the participants

Among the participating nurses, 92.3% were female and the average age was 28.62 years. The majority were married (81.4%) and staff nurses (89.1%). Their average work experience was 63.87 months, and 33.3% had a career of 1–3 years. Most nurses had a bachelor’s degree or higher (86.5%) and worked eight-hour shifts (80.1%; Table 1).

### Differences in variables based on demographic characteristics

There were significant differences in burnout depending on gender, years of work experience, current position, and shift work. Specifically, participants with a work experience of more than 3 years but less than 5 years had a higher perception of burnout than those with less than 1 year of work experience (F = 4.063, *P* = 0.004). Women had a higher perception of burnout than did men (t = 3.439, *P* = 0.001), and staff nurses had a higher perception of burnout than did charge nurses (t = 2.339, *P* = 0.021). In addition, participants who worked eight-hour shifts had a higher perception of burnout than did those who worked 12-hour shifts (t = 2.204, *P* = 0.029; Table 1).

### Participants’ burnout, WFC, and sleep disturbance

Table 2 presents the means and standard deviations of burnout, WFC, and sleep disturbance. The mean score of burnout was 78.82 ± 10.77. The mean scores of WFC and sleep disturbance were 28.82 ± 6.87 and 71.02 ± 16.68, respectively.

### Correlation among WFC, sleep disturbance, and burnout

As indicated in Table 3, burnout was positively correlated with WFC (*r* = 0.224, *P* = 0.005) and sleep disturbance (*r* = 0.500, *P* < 0.001).

### Mediating effect of sleep disturbance on the association between WFC and burnout in nurses

**Fig. 1:**
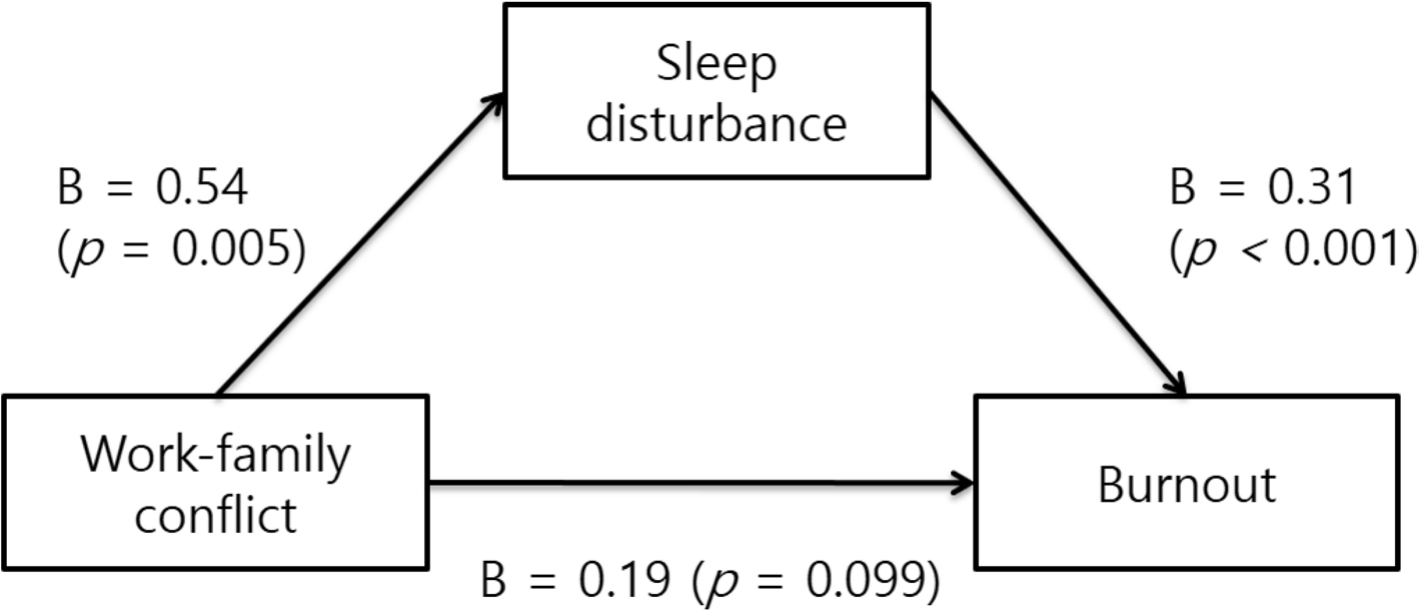
Mediating effect of sleep disturbance on the association between work–family conflict and burnout in nurses

PROCESS Macro Model 4 was used to test whether sleep disturbance mediated the association between WFC and burnout. The results showed that WFC was not directly related to burnout (b = 0.19, 95% CI [− 0.03, 0.20]), but was indirectly related to burnout through sleep disturbance (b = 0.17, 95% CI [0.01, 0.32]). That is, WFC did not directly affect burnout, but exerted a significant indirect effect via sleep disturbance (Table 4, Fig. 1).

## Discussion

To the best of our knowledge, no studies have examined the association among WFC, sleep disturbance, and burnout in Korean nurses. The results of the present study confirmed that nurses’ perception of sleep disturbance mediates the effect of WFC on burnout.

In our study, Korean nurses experienced above-average burnout, which was higher than that reported by previous cross-sectional studies conducted with nurses [9, 21]. In a previous study, nurses’ burnout in China was higher than that in many Western countries, indicating that it is a prevalent problem in developing countries [24], supporting our findings. Therefore, nurse managers should focus on reducing and preventing burnout.

In our study, women had higher burnout than did men. Inconsistent with our study results, some studies do not show a difference in burnout by gender [6]. The proportion of male nurses is about 5% in Korea, 12% in the United States [25], 9% in Canada [26], and 8% in New Zealand [27]; that is, the proportion of male nurses worldwide is low. Similarly, male nurses accounted for 7.7% of our study sample, indicating a significant difference when compared to the ratio of female nurses; this implies that caution should be exercised when generalizing our findings on gender differences in burnout. Nevertheless, research shows that Korean male nurses experience burnout and change their jobs as a result of being rejected by patients or guardians [28], a lack of belonging to the nursing department, and limitations in promotion [29]. These burnout triggering factors differ from those found in female nurses, necessitating additional research into gender differences in burnout.

Furthermore, unmarried nurses had significantly higher burnout scores than married nurses. This could be because the organization expects married nurses to perceive similar situations more positively than unmarried nurses due to family support [30], and assumes that unmarried nurses can handle higher workloads than married nurses with children and families [31].

In this study, burnout was found to be higher among nurses who worked 8- rather than 12-hour shifts. Partially supporting our findings, a study conducted with Italian nurses found that those working 8-hour shifts had higher burnout than their 12-hour shift counterparts [14]. The difference may be due to a divergence in the number of shifts rather than a difference in working hours (i.e., the 8-hour shift being shorter than the 12-hour shift). The eight-hour shift increases the number of shifts of nurses due to the three types of work that they can undertake (day, evening, and night). This increased burnout among those who work eight-hour shifts is presumed to be due to high fatigue caused by irregular lifestyles and changes in the circadian rhythm [32]. Meanwhile, a study conducted with North American police officers showed that burnout increased with long shifts (more than 11 hours) [33]; a study on nurses in Iran showed that there was no relationship between burnout and shift work of nurses [34]. As the literature provides inconsistent data on the effects of job-related variables on burnout in nurses [1], additional studies are required to reveal the factors affecting burnout.

In this study, nurses’ burnout showed a positive correlation with WFC and sleep disturbance. Studies in Canada [26] and Italy [11] also showed that nurses’ WFC was correlated with burnout; research in Taiwan [10] and China [12] demonstrated that the higher the WFC of nurses, the significantly greater their sleep disturbance. Furthermore, a study found that WFC affects sleep quality and disturbance [9]; all these cited studies support our findings.

Moreover, Kexian et al. [12] reported that the higher the family–work conflict (FWC), the shorter the sleep duration; nevertheless, there are some limitations in comparing the results of Kexian et al.’s study and our findings. This is because whereas WFC refers to when one’s work-related roles interfere with one’s ability to perform family roles, FWC refers to when one’s home-related roles interfere with one’s ability to perform work roles [35]. In WFC, the creation of a workplace environment in which people can experience job satisfaction while working (e.g., workplace culture improvements, work innovation incentives, and family care support) can help alleviate burnout [30]. In FWC, workplace environments that enable workers’ families to recognize the job (e.g., flexible working systems) can relieve burnout [36]. As the inducing and mitigating factors of FWC and WFC are different, future researchers could analyze the effects of FWC and WFC in nurses to provide an enhanced understanding of the matter.

Regarding practical implications, our evidence generally highlights that nursing managers in Korea should pay attention to WFC, particularly to its influence on sleep and negative effect on sleep quality; they should also focus on department members’ sleep disorders and come up with plans to adjust work schedules while considering individual characteristics and conditions. Prior research demonstrated that changes in work schedules within a short period of time, longer overtime hours, more night shifts, and weekend shifts had a negative impact on WFC [37]. Considering these delineations, improving various elements of the workplace environment of nurses to assist in the reduction of WFC may be necessary, such as introducing a long-term shift work system, securing adequate staffing, and providing institutional and policy support (e.g., improving the shift work system by assigning night workers).

As remarked, women have traditionally played a central role in housework and childcare in Korea, which entails that family support is an important aspect of sleep problem management and improving the sleep quality of female nurses. Accordingly, interventions on sleep for female nurses could consider the involvement of the family. In addition, as the majority of Korean nurses are women, the responsibility for reducing the stress associated with childbirth and child-rearing among female nurses, as well as promoting work-family balance, should not be confined to the individual or the family. Instead, stakeholders could take responsibility for minimizing these issues and develop practical and effective institutional policies (e.g., parental leave and flexible working systems) to help nurses participate in family life while minimizing the impact of WFC on their work.

Our findings showed that both WFC and sleep disturbance affect burnout and that improvements in sleep disturbance may reduce the effect of WFC on burnout. The evidence in the literature support the results of our study, with one research showing that the WFC of hospital nurses influenced perceived health status, and sleep disturbance had a mediating effect on this association [10]. Seeing that burnout negatively impacts both employees and organizations, preventive measures to reduce the impact of risk factors of burnout may be warranted; specifically, our results point to the need for interventions aimed at reducing the effects of WFC and sleep disturbances among hospital nurses. Therefore, hospital managers should be empathetic when scheduling shifts for hospital nurses, as this action may help nurses maintain their sleep quality, thereby reducing the health impacts of WFCs.

### Limitations

This study has some limitations. First, it was conducted in only one secondary hospital in a single city in Korea, and all participants were Korean; because of Korea’s unique cultural characteristics, generalizations at the international level should be made with caution. Second, no comparative analysis was performed according to nurses’ work type or the presence of children at home. Third, no distinction was made between the effect of the work and personal life of nurses on their burnout. Fourth, this study was conducted during the COVID-19 pandemic; ever since its onset, nursing staff have been suffering from fatigue due to increased workloads and significant WFCs. Thus, once more, we highlight that caution should be exercised when interpreting or generalizing the study results. Based on these limitations, we acknowledge the need for follow-up studies on the effect of children’s presence at home and shift schedule on sleep disturbance. In addition, as a study on factors affecting burnout of nurses, it is necessary to separate and analyze work and personal life at work.

## Conclusions

We expanded the existing evidence on WFC and burnout among nurses by revealing the mediating effect of sleep disturbance. Specifically, it was confirmed that sleep disturbance had a complete mediating effect on the relationship between WFC and burnout of nurses. Therefore, burnout prevention strategies should focus on dealing with nurses’ sleep disturbances in order to mitigate the effect of WFC on burnout. Our findings can be used as reference data for developing an efficient system for improving nurses’ sleep health and designing appropriate working schedules.

## Data Availability

Data cannot be shared publicly because of restrictions imposed by the Konyang University Institutional Review Board. Data are available from the Konyang University Institutional Data Access/Ethics Committee for researchers who meet the criteria for access to confidential data. Data requests can be addressed to the Konyang University Institutional Review Board (82–42–600-8466, kirb@konyang.ac.kr).

## Abbreviations

CI: Confidence interval
COPSOQ II: Copenhagen Psychosocial Questionnaire II
COVID-19: Coronavirus disease-19
WFC: Work–family conflict
FWC: Family–work conflict
